# Maximizing Interpretability and Cost-Effectiveness of Surgical Site Infection (SSI) Predictive Models Using Feature-Specific Regularized Logistic Regression on Preoperative Temporal Data

**DOI:** 10.1155/2019/2059851

**Published:** 2019-02-19

**Authors:** Primoz Kocbek, Nino Fijacko, Cristina Soguero-Ruiz, Karl Øyvind Mikalsen, Uros Maver, Petra Povalej Brzan, Andraz Stozer, Robert Jenssen, Stein Olav Skrøvseth, Gregor Stiglic

**Affiliations:** ^1^Faculty of Health Sciences, University of Maribor, Maribor 2000, Slovenia; ^2^Department of Signal Theory and Communications, Telematics and Computing, Universidad Rey Juan Carlos, Fuenlabrada 28943, Spain; ^3^UiT Machine Learning Group, UiT the Arctic University of Norway, Tromsø 9037, Norway; ^4^Department of Mathematics and Statistics, UiT the Arctic University of Norway, Tromsø 9037, Norway; ^5^Faculty of Medicine, University of Maribor, Maribor 2000, Slovenia; ^6^Faculty of Electrical Engineering and Computer Science, University of Maribor, Maribor 2000, Slovenia; ^7^Department of Physics and Technology, UiT the Arctic University of Norway, Tromsø 9037, Norway; ^8^Norwegian Centre for E-health Research, University Hospital of North Norway, Tromsø 9037, Norway

## Abstract

This study describes a novel approach to solve the surgical site infection (SSI) classification problem. Feature engineering has traditionally been one of the most important steps in solving complex classification problems, especially in cases with temporal data. The described novel approach is based on abstraction of temporal data recorded in three temporal windows. Maximum likelihood L1-norm (lasso) regularization was used in penalized logistic regression to predict the onset of surgical site infection occurrence based on available patient blood testing results up to the day of surgery. Prior knowledge of predictors (blood tests) was integrated in the modelling by introduction of penalty factors depending on blood test prices and an early stopping parameter limiting the maximum number of selected features used in predictive modelling. Finally, solutions resulting in higher interpretability and cost-effectiveness were demonstrated. Using repeated holdout cross-validation, the baseline C-reactive protein (CRP) classifier achieved a mean AUC of 0.801, whereas our best full lasso model achieved a mean AUC of 0.956. Best model testing results were achieved for full lasso model with maximum number of features limited at 20 features with an AUC of 0.967. Presented models showed the potential to not only support domain experts in their decision making but could also prove invaluable for improvement in prediction of SSI occurrence, which may even help setting new guidelines in the field of preoperative SSI prevention and surveillance.

## 1. Introduction

Surgical site infections (SSIs) are the most common type of nosocomial infections and a major cause of morbidity among surgical patients, especially following abdominal and colorectal [1], cardiovascular [2], oncological [3], and trauma or orthopaedic surgeries [4, 5]. An empirical surveillance study [6] for patients undergoing surgical procedures (SP) that encompassed data from 82 hospitals in 30 countries confirmed that the highest SSI rates from SP were from the aforementioned surgeries, where ventricular shunt had the highest rate with 12.9%, followed by colon surgery with 9.4%, bile duct, liver, or pancreatic surgery with 9.2%, abdominal aortic aneurysm repair with 7.7%, and thoracic surgery with a 6.1% rate.

The best strategy in SSI prevention lies in effective guidelines to the issue of SSI prevention, and most are specifically tailored to meet the needs of the countries in which they were published [7]. The World Health Organization (WHO) in 2016 published global guidelines on the prevention of SSI [8], which included conducting specific tests per patient prior to a certain medical procedure. There are several approaches to predict occurrence and reduce the incidence of SSIs, such as different risk models and prevention strategies. For instance, high-income countries have realized that collecting data through centralized surveillance systems is an essential component of SSI prevention [7, 9–11]. Risk models aim at predicting SSIs and guiding further action to prevent more serious outcomes.

In addition to the health risk for the patient, SSI usually implies longer postoperative hospital stays, considerably increased postoperative costs, and often a higher mortality (on average by 9.7 days with an increased cost of $20,842 per admission) [12]. The economic costs alone of SSIs are substantial [13], for example, when looking from the national perspective (in the US), the SSI cases were associated with additional 406,730 hospital-days and hospital costs exceeding $900 million. An additional 91,613 readmissions for treatment of SSI accounted for a further 521,933 days of care at a cost of nearly $700 million [14].

Several researchers have already focused on predicting SSI based on electronic health records (EHRs), for example, by using patient demographics, past medical history, and surgical information [15]. In recent studies, ranging from model evaluation methods, such as ROC analysis [16, 17], to data-driven modelling approaches, such as linear regression models [1, 18, 19] and Support Vector Machines [20], the attention has shifted to results of blood tests before and after surgery. One of the main reasons being the ease and extent of this type of data. Specifically, blood test results of C-reactive protein (CRP) have been associated with a high predictive power [1].

Interpretability of the current practices and models therefore starts with CRP, a known indicator of inflammation, and is the first in line of predictors associated with the presence of inflammation of any kind, but it also has its drawbacks. Among these is the fact that if a high CRP is measured in a patient prior to surgery, the surgery will most likely get postponed. Consequently, CRP is not a predictor, but more likely a filter of having a surgery in a specific patient at all. However, it is of course also clear that if surgery gets underway in a patient with an already high mean CRP value, the probability that the surgical site gets infected increases.

Several studies have taken a classical (knowledge-based) approach and handcrafted features by setting cutoff values of CRP after the surgery, where the term of postoperative day (POD) is commonly used. For example, Angiolini et al. [19] evaluated the diagnostic accuracy as an early predictor of SSIs after pancreaticoduodenectomy and showed that CRP on POD 3, with a cutoff of 17.27 mg/dl, predicted the postoperative course in 78.2% of patients, whereas a CRP cutoff of 14.72 mg/dl on POD 4 predicted the postoperative course in 80.2% of patients. A systematic review of studies on diagnostic value of CRP after major abdominal surgery for predicting SSI [21] showed that CRP > 15.9 mg/dl on POD 3 increases the risk of SSI [10].

An alternative data-driven approach for the SSI problem was demonstrated by Ke et al. [22], where the focus was on dynamic wound data (e.g., using mHealth tools, which include self-reported symptoms of pain, body temperature, wound features, and patient- or caregiver-generated images of the wound) for SSI prediction. Predicting time to SSI onset with spatial-temporal data via bilinear formulation was used and further enhanced with automatic missing data imputation by the matrix completion technique for data from POD 2 until discharge or POD 21, whichever was earlier. This approach showed superior performance on real-world data set of SSI in terms of mean absolute error (MAE) compared to linear regression and support vector regression (SVR) [22]. The aforementioned studies have all focused on postoperative data. However, as Silvestre et al. [18] noted, preoperative CRP concentrations were significantly higher already prior to surgery in patients that developed infections postoperatively than those who did not develop complications. This suggests that there is a high risk that an underlying infection was already present prior to surgery. Hence, if we detect those patients prior to surgery, preventive interventions can be performed, minimizing the risk of SSI. We believe that a data-driven approach that alleviates the vast amounts of information stored in the EHRs, and in particular in blood samples, is valuable for predicting SSI prior to surgery. Such an approach already achieved remarkable success rates at predicting development of SSIs following gastrointestinal surgery [20].

Predicting SSI after surgery gives us additional intra- and postoperative risk factors such as surgery duration [23–25], treatment complexity [25], blood loss during surgery [24], administration of supplemental oxygen [26], and higher intraoperative lactate levels [23] which in turn can improve the SSI prediction or augment an existing preoperative data SSI prediction.

We further summarized our findings of studies focusing on pre- and/or postoperative data in the prediction of SSI in Table 1, where we compared tasks, data, and methods used.

We conjecture that a higher predictive power lies in the expansion of the preoperative blood test results from the mean value to more complex parameters (e.g., slope of linear regression line for a fixed temporal window and number and proportion of low/high abnormal values of tests for a fixed temporal window). This presents a unique approach, which was to our best knowledge, not yet used in SSI prediction.

Considering the available models for predicting SSI based on preoperative data, our motivation was to improve their predictive potential, while at the same time maximizing their interpretability and final diagnostic cost-effectiveness. As such, this study presents a novel approach to SSI prediction among patients undergoing gastrointestinal surgery based on temporal data from blood test results. Our solution is built on the abstraction of preoperative blood test data in three different temporal windows. In order to maintain interpretability, penalized logistic regression was used as a core classification method since it allows tuning of the model complexity to avoid overfitting and still maintains a high level of predictive performance. Moreover, clinical knowledge was incorporated into the model, and also features that account for the amount of missing data were proposed. Additional solutions were developed to produce a more cost-effective model by introducing a penalization based on the price of the respective blood tests. Using this approach, it is possible to achieve better economic efficiency of the models in practice as our approach aims to reduce the costs of laboratory tests by eliminating more expensive tests in cases where similar results can be achieved by combining tests costing less.

## 2. Materials and Methods

### 2.1. Data Set Description

The data used in this work was previously explored and analyzed by Soguero-Ruiz et al. [20]; further, it was used in the American Medical Informatics Association Knowledge Discovery and Data Mining 2016 data competition. The data set we considered consists of 7725 patients that underwent a gastrointestinal surgical procedure at the University Hospital of North Norway in the years 2004–2012. Since SSI-persistent in-hospital morbidity is particularly associated with colorectal cancer surgery [33], patients who did not undergo this type of surgery were excluded, reducing the size of the cohort to 1137 patients. Guided by input from clinicians, the International Classification of Diseases (ICD10) and NOMESCO Classification of Surgical Procedures (NCSP) codes related to severe postoperative complications, and in particular to SSI, were considered to identify patients with SSI. Patients who did not have these codes or the word “infection” in any of the postoperative text documents were considered as controls. 80% of the cohort (909 patients) was used for model development, and the remaining 20% of the cohort was set aside for model testing. In the model development set, 183 out of 909 patients (20.13%) developed SSI, whereas in the test set 50 out of 228 (21.93%) patients developed SSI.

Data included information from various blood tests (811 different blood tests at different points in time). The data ranging from preoperative day 5393 up to the day of surgery were used, while data collected postoperatively were not used in this study. For each blood test, the mean number of blood test values recorded in the last 30 days before the surgery was calculated. In total, 14 most frequent blood tests (with a mean number of measurements above one) were used: hemoglobin (5.37 measurements), leukocytes (4.43), sodium (4.24), CRP (4.11), potassium (3.97), albumin (2.87), creatinine (1.94), thrombocytes (1.53), alanine aminotransferase (ALT, 1.23), total bilirubin (1.22), aspartate aminotransferase (AST, 1.14), glucose (1.06), amylase (1.06), and alkaline phosphatase (ALP, 1.04). From a medical point of view, the abovementioned blood tests can have a specific or a more general role in the context of gastrointestinal surgery. ALT, AST, ALP, total bilirubin, and albumin serve to specifically assess hepatic functional capacity, inflammation or biliary tract obstruction, and amylase pancreatic ductal obstruction and inflammation. Glucose is a much more general metabolic marker, depending heavily on insulin sensitivity in peripheral tissues, but may be of special relevance in gastrointestinal patients with liver or pancreatic disease, due to deranged central insulin sensitivity or reduced gluconeogenesis, and diminished insulin secretion, respectively. The remaining 7 tests are even less specific for gastrointestinal conditions. Reduced hemoglobin may indicate chronic gastrointestinal bleeding, elevated CRP, and leukocytes inflammation. Sodium, potassium, and creatinine levels help to assess water and electrolyte balance and kidney function, and thrombocytes the risk for bleeding or thrombosis [34–36].

Some of these represent parameters obtained in routine blood tests, while others are more specifically aimed at detecting inflammation or infection, making them easier to interpret in the context of SSI model prediction.

### 2.2. Feature Representation

Following an initial exploratory analysis of model development data set using different visualization techniques (observing different patterns with regard to SSI), we set the observation interval to 60 days before the surgery for all selected blood test based features. The initial data set of feature representation thus consisted of 14 most frequent blood tests for an interval of 60 days before surgery on a daily basis, where the mean values of blood tests were used if more than one value was recorded in a day. Since all tests were not available on a daily level, with a large percentage of missing values ranging from 90.25% for hemoglobin to 98.19% for amylase present, imputation using three approaches was used: (a) last observation carried forward (LOCF) [37], (b) *k*-nearest neighbours (KNN) [38], and (c) a combination of LOCF and KNN, where LOCF was used for the farthest time point, i.e., 60 days before surgery and KNN elsewhere. The last approach enabled us to benefit both from the addition of patient history for patients who did not have certain blood tests 60 days before surgery with LOCF and the imputation of results based on the nonmissing values of the neighbours with KNN.

**Figure 1:**
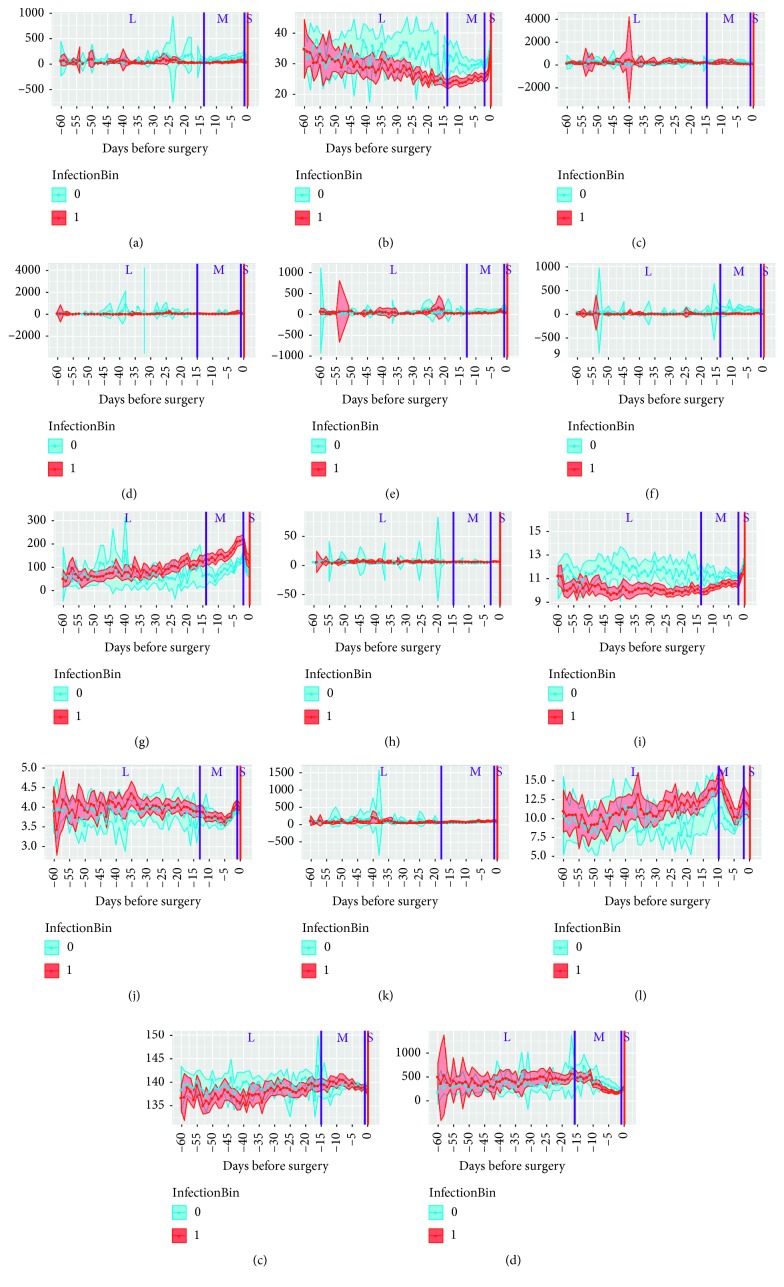
Selection of time windows based on visualization of 14 parameters for 909 patients during 60 days before the surgery. (a) ALAT. (b) Albumin. (c) ALP. (d) Amylase. (e) ASAT. (f) Bilirubin total. (g) CRP. (h) Glucose. (i) Hemoglobin. (j) Potassium. (k) Creatinine. (l) Leukocytes. (m) Sodium. (n) Thrombocytes.

Additionally, three temporal windows (S, short; M, medium, and L, long) were manually defined for each feature based on the observed patterns (e.g., peaks or changes in trends) of feature values prior to surgery in (Figure 1). The S window included measurements from days 2 to 0 (depending on observed pattern for specific blood test) prior to surgery, in which the most tests per day were performed. The M window included data from days 18 to 13 prior to surgery up to the start of the S window. The L window encompassed the period from preoperative day 60 to the upper limit of window M.

Since the length of the observation interval could influence the SSI prediction results, we included shorter observation intervals, more specifically a 30-day and 15-day observation interval. In the 30-day observation interval, the L window encompassed a shorter temporal window from preoperative day 30 to the upper limit of window M. The 15-day observation interval had 1 temporal window from 15 to 0 days before surgery.

While changes in blood test values in the short temporal window can be associated with acute infections in patients prior to surgery (that can clearly lead to SSI after surgery), the longer temporal windows (medium and long) are more indicative of some underlying (maybe even not properly treated) pathophysiological changes that could have an indefinite influence on the SSI outcome. Looking at the parameters that were chosen by our model to predict SSI, it is clear that the different temporal windows play an important part in the battery of predictors for SSI.

The following features were extracted for each temporal window separately:

- Mean value for each set of blood test measurements.
- Slope of the simple linear regression for each set of blood test measurements (representing the trend).
- The number of measurements for each blood test (calculated before imputation).
- Proportion of measurements in current window, calculated as the number of measurements in current window divided by the number of all available measurements in 60, 30, or 15 days before the surgery for each patient (calculated before imputation).
- Number of abnormal (high and low) blood test values for each blood test as defined in Norwegian National Guidelines [39] (calculated before imputation).
- Proportion of high/low abnormal values, calculated as the number of high/low abnormal values for each blood test divided by the number of all blood test values recorded in different temporal windows (calculated before imputation). These features were introduced as an early indicator for developing SSI, with our underlying assumption that patients with a higher proportion of abnormal blood tests develop SSI more frequently.

Since there were 14 blood tests and 3 temporal windows available for each of the three types of generated features, with 6 features generated in each instance, the final datasets consisted of 252 features for the 60-day and 30-day observation interval and 84 features for the 15-day observation interval.

### 2.3. Predictive Modelling

As maximizing interpretability was one of our goals, we restricted modelling to linear models with an additional model based on an ensemble of boosted decision trees serving as a nonlinear comparison. We further restricted linear models to regularized linear models allowing the complexity of the model to be tuned for a better predictive performance, thus avoiding overfitting.

A generalized linear model via penalized maximum likelihood L1-norm (lasso) regularization was used as defined by Friedman et al. [40]:

(1) $$\begin{array}{c} \underset{\beta_{0},\beta }{\min}\frac{1}{N}\overset{N}{\underset{i=1}{\sum }}w_{i}l\left(y_{i},\beta_{0}+\beta^{T}x_{i}\right)+\lambda \beta_{1}, \end{array}$$

where *i* represents observations and its negative log-likelihood contribution is noted as *l*(*y*, *η*), *w*_*i*_ noting weights and tuning (shrinkage) parameter *λ* controlling the overall strength of the penalty. We excluded a broader elastic net regularization, as it did not show any significant gain in our initial experiments and added complexity to the model, making it less interpretable.

Due to the class imbalance with only 20.13% positive cases in the development set, random oversampling examples (ROSE) [41] technique was used.

Additionally, a prior knowledge of predictors (blood tests) was integrated in the modelling by introduction of penalty factors for each coefficient *β*_*j*_, *j*=1,…, *p*, which depended on blood test prices (these vary from test to test, with the most expensive costing twice the price of the cheapest one; Table S1). The penalty term can be described as minimizing the coefficients *β*_*j*_, *j*=1,…, *p* in the following equation:

(2) $$\begin{array}{c} \lambda \overset{p}{\underset{j=1}{\sum }}v_{j}P_{\alpha }\left(\beta_{j}\right)=\lambda \overset{p}{\underset{j=1}{\sum }}v_{j}\left|\beta_{j}\right|, \end{array}$$

where *v*_*j*_ represents the penalty factor of coefficient *j*.

An additional user-defined parameter *p*_max_ was used in the above-described framework to limit the parameter *λ* in a way that the maximum number of selected features in the model cannot exceed *p*_max_. This parameter can be seen as an “early stopping” parameter, as it stops the *λ* cross-validation tuning as soon as the number of selected features exceeds *p*_max_, thus providing a much higher level of interpretability and generalizability of the model. To compare results with a nonlinear-based solution, an optimized tree learning-based distributed gradient boosting framework called XGBoost [42] was used with the full set of 252 features. Selection of parameters for it was done by selecting the best performing set of values of parameters via 100 repeated evaluations of random range values for parameters on fixed training and validation set. It has to be noted that ensemble-based models are less interpretable and are not preferred in cases where model explanation can be of practical use (in our case for clinician treating the GIT surgery patient or extracting new knowledge that could lead to new guidelines).

### 2.4. Cost-Efficient Feature Penalization

The prices for each blood test were obtained from the Department of Medical Biochemistry, Oslo University Hospital. The blood tests with lower prices were assigned lower feature-specific penalization coefficients *v*_*j*_ that were calculated as *v*_*j*_=*r*_max_/*r*_*j*_, with *r*_max_ representing a price of the most expensive blood test (leukocytes and thrombocytes at 58 NOK) and *r*_*j*_ representing a price of the blood test *j*. The lowest value of *v*_*j*_ was calculated for a group of glucose blood test-related features (23 NOK).

## 3. Results

### 3.1. Experimental Setup

Repeated holdout cross-validation approach on model development data set, using 80% of data for training and 20% for validation, was used in order to ensure the generalizability of the predictive model results. The holdout cross-validation was repeated 100 times in each experiment to obtain mean values and 95% confidence intervals (CI) for each performance metric (Table 2). The following widely used performance metrics were used in all experiments (Tables 2 and 3): area under the ROC curve (AUC) as primary evaluation metric; area under the precision recall curve (AUPRC) as a secondary evaluation metric, since it summarizes the PPV (i.e., ratio of correctly classified positive values to the number of all instances classified as positive) over sensitivity into one number and it can be often more informative than AUC in cases of unbalanced datasets [43], threshold, sensitivity, specificity, positive predictive value (PPV), and negative predictive value (NPV).

The same performance metrics were also used on the test set (*n*=228) to evaluate the final model built on the model development set (*n*=909).

### 3.2. Results Using Penalized Logistic Regression

Initially, experiments using L1-penalized logistic regression (lasso) were conducted without imposing a limit to the number of selected features *p*_max_ in the *λ* cross-validation tuning step, with different imputation methods and different observation intervals. The results showed that when we used different imputation methods for different observation intervals (60, 30, or 15 days), the three imputation methods (LOCF, KNN, and the combination of LOCF and KNN) performed similarly, more precisely AUC, AUPRC, and PPV were generally less than 1% apart same observation intervals and considering that the combined LOCF and KNN imputation method often performed better in PPV we chose to be used in further experiments. The results for LOCF and KNN imputation for the 15-day observation interval with 1 temporal window resulted in an AUC of 0.937, AUPRC of 0.797, sensitivity of 0.832, specificity of 0.893, and PPV of 0.668; the 30-day observation interval with 3 temporal windows resulted in an AUC of 0.951, AUPRC of 0.812, sensitivity of 0.852, specificity of 0.890, and PPV of 0.665; and the 60-day observation interval with 3 temporal windows resulted in an AUC of 0.952, AUPRC of 0.810, sensitivity of 0.859, specificity of 0.899, and PPV of 0.684. We selected the 60-day observation interval with 3 temporal windows with LOCF and KNN imputation for further experiments since AUC, AUPRC, and PPV values were the most balanced.

These experiments were followed by experimental runs with different *p*_max_ values ranging from 10 to 100 in steps of 10 to find the best balance between the interpretability and predictive performance of the model. When observing the influence of relaxing the restriction on the maximum number of features on the mean AUC, the stabilization of mean AUC was observed with *p*_max_ of 50 (Table 2).

Initial experiments included the use of ROSE algorithm, where we oversampled due to class imbalance of 20.13% positive cases, but there was a loss in terms of AUC on average of 0.5%; therefore, we did not include rebalanced data in further modelling.

The first results (CRP) with an AUC of 0.797 were obtained from a very simple (baseline) model, where only three features representing the mean CRP value in L, M, and S windows were used together with age and sex variables. In the basic lasso model (BLM), only the values of the recorded blood tests were used (mean value, slope, number, and proportion of abnormal values for each time frame interval). Since there were 14 blood tests and 3 time windows available for each of the three types of generated features, the data set consisted of 126 features. Together with age and sex, there were 128 features available to build the BLM. As it can be observed from Table 2, the mean AUC value for the BLM with *p*_max_ restricted to 20 features decreased only slightly, from 0.947 to 0.943, compared to the unrestricted model optimized for maximal AUC.

In the next step, the models were built using additional features (e.g., number and window-specific proportion of blood tests performed in a specific window), where a significant increase in all evaluation metrics can be observed, indicating that the features describing the number and proportion of blood tests in a specific time window represent an important contribution to the model. The mean AUC value of the full lasso model (FLM) increased to 0.954 and was surprisingly slightly higher for *p*_max_ set to 50 to 0.956, whereas AUPRC increased from 0.819 to 0.821 in the same way. This represents a gain (∼1%) in AUC compared to the BLM.

### 3.3. Results Using the Price Penalized Model

The next experiment price penalized model (PPM) aimed at producing a more cost-effective model by taking into account the price of blood tests. When comparing the unrestricted PPM and the model restricted to maximum 20 features, a slight decrease in all evaluation metrics can be observed. Interestingly, also the mean number of features included in unrestricted PPM decreases from 32.6 features in the FLM to 25.2 features in the PPM, which points to the fact that two or more tests might have been replaced by single more-expensive tests. The restriction of the model to a maximum number of features is even more reasonable, although a slight decrease in evaluation metrics can be expected. The highest decrease was observed in PPV, i.e., by more than 2%, and the other metric decreased by less than 1%. However, the mean number of features used in the restricted PPM decreased to 12.1. Additionally, we calculated the provisional costs of all tests that would need to be performed in case of the FLM in comparison to PPM. The costs of all tests recorded for patients in the validation set of 100 cross-validation runs were calculated (simulating 18,100 patients). A marginal cost reduction of only 4.2% was observed in unrestricted FLM vs. PPM. However, in case of limiting the maximal number of features to 20, a significant cost reduction of 48.1% was obtained, reducing the costs for FLM from 25,783,747 NOK to 12,401,982 NOK needed for PPM.

## 4. Results Using Extreme Gradient Boosting of Decision Trees

Finally, the extreme gradient boosting of decision trees was tested on a full set of features. The best results were obtained using an ensemble of 20 decision trees with a maximum depth of 10. The mean AUC value of 0.954 is comparable to the FLM with a mean number of features at 32.6.

### 4.1. Model Selection

However, the FLM was chosen on the basis of lower complexity and higher interpretability. The most frequent features in 100 iterations of the FLM evaluation and their signs of regression coefficients are shown in Table 4. It can be observed that 8 features are included in the model for most of the iterations (above 95%). More precisely, CRP in the medium window is the only feature for which the mean parameter value was selected. This makes sense also from a medical point of view, since CRP is one of the most common indicators of systemic inflammation and is often increased due to infection. Three other selected features from the medium window are related to the number of performed tests (leukocytes and sodium), five features present the proportional number of respective tests (short window hemoglobin, medium window thrombocytes, hemoglobin, and long window leukocytes), and the final one presents the slope of albumin in the long window. The number and proportion of performed tests probably relate to the attending physician's instinct or experience. More specifically, the number of tests for counting leukocytes could indicate the attending physician's assumption of a possible infection.

### 4.2. Model Testing

**Figure 2:**
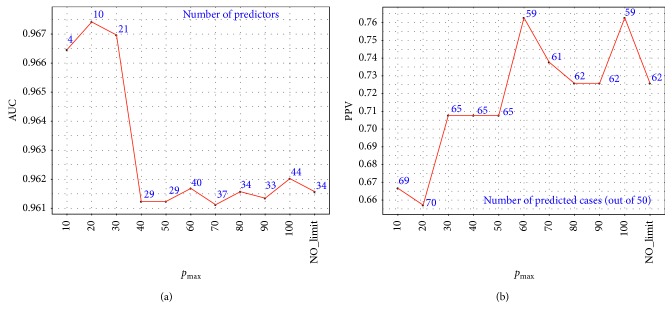
Model testing with regard to parameter *p*_max_ in terms of AUC with number of selected predictors (a) and PPV with number of correctly classified cases out of 50 (b).

The final models, built on the model development data set (*n*=909), were evaluated using the test set (*n*=228) with 50 positive cases for FLM using different *p*_max_ values. The optimal result in terms of AUC/AUPRC was surprisingly at *p*_max_=10 and *p*_max_=20 with some performance metrics better with *p*_max_=10, like AUPRC of 0.882, specificity of 0.971, and PPV 0.667; other were better with *p*_max_=20, like AUC of 0.967 (Table 3). Additionally, it can be seen that, at *p*_max_=20, six more predictors were selected with a total of 10 predictors than at *p*_max_=10 and more positively predicted cases were predicted with a total of 70 (Table 3). A graphical representation of test set evaluation in terms of AUC and PPV at different *p*_max_ values is also shown (Figure 2). When looking at selected features, we can see that four positive predictors were selected in all models: hamoglobin and leukocytes number of test in the medium window, potassium number of tests in the medium window, and thrombocytes proportional number of tests in the medium window.

## 5. Discussion

Norway has one of the highest rates of SSIs in the gastrointestinal tract (GIT), which is consistent with a very high incidence of colorectal cancer that is the most common cause of GIT surgery [12]. Our results support the practical applicability of a combination of blood test results and the temporal testing pattern in predicting SSIs in a clinical setting. More specifically, in addition to the values of a given blood test per se (e.g., CRP), our findings demonstrate that additional extracted features (e.g., number and window-specific proportion of blood tests performed in a specific window) can be very informative with regard to predicting SSIs.

We are aware that the manual selection of temporal windows may have introduced some bias in evaluation of the solutions and is a limitation of the study, but we believe that the sample is big enough to reflect the trends present also in the test set. Future work will improve the selection process with an automatic selection process. One general algorithm considered is the maximum distance between windows approach. A two-step approach, where we firstly select the best partitioning on *k* temporal windows in terms of the highest score function and secondly we repeat the first step on all different sizes of partitionings, which gives us the optimal partitioning. More precisely, we define a distance function for differentiating of daily mean values curves with CI between patients who develop SSI and those who do not for a fixed window length of a selected blood test, for example, *d*_*ij*_ would note a distance function between days *i* and *j*. Next, we define a *k*-partitioning of the whole window length as *S*_1_,…, *S*_*k*_ for *S*_1_ ∪ …∪*S*_*k*_=Ω, *S*_*i*_∩*S*_*j*_=∅,  *i* ≠ *j* and the score function for this partitioning as *f*(*d*_*S*_1__,…, *d*_*S*_*k*__), where the *f* function could be as simple as multiplication of distance functions. For all the *k*-partitionings of a fixed *k* ≤ *n*, we then select the *k*-partitioning with the highest score function value. The second step is using the first step for each of 1 ≤ *k* ≤ *n* partitionings, giving us the optimal partitioning. One drawback of such an approach could be computational intensity, since for *n* = 60-day window and *k*=3 partitions, a total of 34,220 partitions of a specific blood test are possible.

Since testing is mostly guided by judgment of clinicians, which is in turn based on patient history, clinical signs, previous blood tests, results of diagnostic imaging, general patient observation etc., we believe that our choice to include information on testing patterns enabled us to indirectly include judgment by expert clinicians into our predictive model. Moreover, we believe that, due to its intuitive nature, clinicians could easily relate to and accept our predictive model.

With regard to values, among all tests, CRP values above normal played a predominant role in our model. From a mechanistic point of view, increased CRP is highly suggestive of an underlying inflammatory process. Our findings indicate that, also in the period preceding operation, a higher-than-normal CRP value raises the probability that a patient will develop SSI. The mechanistic substrate for this observation might be that the underlying inflammatory process indicates an increased susceptibility toward infections or even contributes to their development.

Recently, it has been suggested that signs of preoperative inflammation may predict postoperative infectious complications, specifically in patients undergoing colorectal surgery [30, 44, 45]. At least 4 out of our 6 positive predictors may be viewed as markers of inflammation, namely, the mean CRP value in the medium window, leukocytes with high proportion of abnormal values/number of tests in the long window, and leukocytes number of tests in the medium window. Local inflammation impairs the healing process, and systemic inflammation interferes with the immune response. The fact that our positive predictors pointed to inflammation or a suspected inflammation in the long or medium period might suggest that, during this time, they were a better marker of chronic inflammation than just shortly before the procedure. The predictive roles of thrombocytes and potassium remain to be confirmed and explained in future studies. However, it is tempting to speculate that low thrombocyte counts may directly impede the healing process, whereas high numbers may indicate or even modulate inflammation [46], and that the number of potassium concentration tests reflects more fragile patients with disturbed water and electrolyte homeostasis, e.g., due to abnormal ADH secretion or kidney disease.

Our study included only GIT surgery patients, mainly due to the high risk of SSIs in this group [6]. It is reasonable to speculate that a similar predictive model could be developed and used for other types of surgeries. A recent study indeed found that CRP, together with preoperative levels of albumin, hemoglobin, and signs of mild or moderate kidney failure, was significantly associated with the odds of SSI in a diverse group of patients undergoing general, oncologic, trauma, and vascular surgery procedures [32]. Even so, it remains to be investigated systematically whether in these and other groups of patients, inclusion of information on testing patterns will result in better predictive models.

Let us now discuss the wider importance of studies similar to ours that try to shed more light on the predictive power of preoperative blood tests in prediction of SSI. It is generally accepted that effective guidelines are key in prevention of SSI. On one hand, clinical guidelines pave the way for specific tests that need to be conducted in a certain patient prior to a specific surgery. On the other hand, the demand for specific tests, which are not always even conducted by the hospital where the patient is hospitalized, mandates the overall cost for a battery of tests. In the case of preoperative blood tests, of which none costs more than 2€ per measurement per patient, we can easily calculate that even a battery of 10 tests conducted three times prior to surgery (which in most cases costs at least a couple of 1000€, depending on surgery type) would cost only 60€. This means we can probably perform as many blood tests as we want and still provide a cheaper solution for the insurance companies (and hospitals) compared to complications and increasing costs connected with SSI. On the other hand, by preventing SSI preoperatively, we at the same time also prevent unnecessary patient complications, the lowered quality of life, and finally, prolonged stays of the respective patients' socioeconomic environment. Longer hospitalization times on one hand tend to induce longer socioeconomic reintegration times, especially in the elderly, while on the other hand, they lead to significantly increased overall treatment costs.

## 6. Conclusions

Our solution is based on abstraction of preoperative blood test data and use of L1-penalized logistic regression to predict SSI. Additional solutions were developed to improve the model's practical interpretability (for the treating clinician as well as for the medical informatician) and to include the cost-effectiveness aspect. The model with the best solution clearly indicates that a specific and easy interpretable SSI-related feature (CRP) has to be paired with rather indirect features related to the available temporal data (number, proportion of tests, etc.). In our opinion, this nicely captures the “treating clinician's suspicion” based on the overall patient evaluation over the course of the period preceding surgery. The model that included CRP only resulted in an AUC of ∼80%, whereas models that additionally included the indirect features reached an AUC of ∼95%.

Considering the high incidence of SSI (especially related to GIT surgery) and the continuous efforts of EU officials to decrease this value, our model could contribute to new guidelines in the field of preoperative SSI prevention and surveillance.

## Figures and Tables

**Table 1:** Summary table of articles focused on pre- and/or postoperative data in the prediction of SSI.

Article	Task	Data	Preoperative/postoperative	Method
Medina-Fernández et al. [1]	Use of CRP identifies postoperative infectious complications in patients undergoing colorectal surgery	Blood tests: CRP, neutrophil-to-lymphocyte ratio (NLR), and white blood cell (WBC)	Postoperative (2 POD)	Multiple linear regression

Dutta et al. [16]	Examine WCC, albumin, and CRP following esophagogastric cancer resection as a predictor of postoperative surgical site infectious complications	Blood tests: White cell count (WCC), albumin, and CRP	Postoperative (7 POD)	Friedman test and Wilcoxon signed-rank test on medians and ranges

Platt et al. [17]	Analyze postoperative WCC, albumin, and CRP and their diagnostic accuracy in case of infectious complications.	Blood tests: WCC, albumin, and CRP	Postoperative (7 POD)	Friedman test and Wilcoxon signed-rank test on medians and ranges

Silvestre et al. [18]	Assess the value of serum CRP and PCT time course in the postoperative setting of elective colorectal surgery with primary anastomosis and its potential in detecting infectious postoperative complications.	Blood tests: CRP and procalcitonin (PCT)	Pre- and postoperative (9 POD)	Student's *t* test, Mann–Whitney *U* test, logistic regression

Soguero-ruiz et al. [20]	Prediction of SSI with individual blood tests and in a joint model considering linear and nonlinear classifiers, both before and after surgery	Blood tests: hemoglobin, leucocytes, CRP, potassium, sodium, creatinine, ALAT, thrombocytes, albumin, and ALP	Preoperative (20 days) and postoperative (20 POD)	Gaussian process, linear and nonlinear SVM

Gans et al. [21]	Systematic review			

Ke et al. [22]	The use of dynamic wound data for SSI risk prediction is explored, by exploiting the low-rank property of the spatial-temporal data via the bilinear formulation	Procedure related data as well as clinical data such as smoking, diabetes mellitus, or alcohol use, among others	Postoperative (21 POD)	Bilinear prediction model, projected gradient descent, bounded matrix completion

Shimizu et al. [27]	The authors investigated the risk factors for SSI in patients who had undergone appendectomy	Blood tests such as CRP, albumin, NLR; the length of the operation, the number of intra-abdominal drains, the term of antibiotic use, the hospital stay, among others; also, clinical background features were considered	Preoperative	Chi-squared test and the Mann–Whitney *U* test; odds ratio (OR)

Ortega-deballon et al. [28]	The aim of this study was to look for a relationship between the fatty tissue metabolism measured by adipocytokine levels and the risk of postoperative infection	Blood tests: preoperative plasma levels of eight adipocytokines, cholesterol, triglycerides, insulin, and CRP; furthermore, patient-specific and intraoperative risk factor for infection such as age and sex, among others	Pre- and postoperative	Chi-squared tests or Fisher's exact tests, Wilcoxon test, Spearman's correlation coefficients, and the odds ratios

Mohri et al. [29]	The aim of this study was to examine the association between postoperative infection and preoperative systematic inflammation in patients undergoing resection of gastrointestinal cancer	Blood tests: white cell count, hemoglobin, albumin, CRP; furthermore, age, sex, tumor site, operative approach, and the American Society of Anesthesiologists (ASA) grade	Pre- and postoperative	Chi-squared tests, Wilcoxon rank test; a multiple logistic regression analysis was also considered

Moyes et al. [30]	The aim was to examine the relationship between the preoperative mGPS (the glasgow prognostic score) and the development of postoperative complications in patients undergoing potentially curative resection for colorectal cancer	Blood tests: white cell count, albumin, and C-reactive protein and clinicopathological characteristics such as age, gender, tumor site, and nodal involvement, among others.	Pre- and postoperative	Mantel–Haenszel (*χ* ^2^) test for trend, logistic regression analysis

Cappabianca et al. [31]	The study objective was to evaluate the effect of CRP on short-term and midterm outcome after cardiac surgery	Preoperative patient profile, including features such as diabetes, body mass index, and smoking history, among others; this type of surgery was also considered	Pre- and postoperative	*χ* ^2^ test, shapiro–Wilk test, kaplan–meier curves, and the log-rank test, logistic regression and cox model

Mujagic et al. [32]	This study examines the association between preoperative biochemical markers and the risk of SSI	Blood tests: hemoglobin, creatinine, albumin, CRP, and white blood cell count; and other baseline features such as ASA and diabetes, among others.	Pre- and postoperative	Fisher's exact test, *t*-test, kruskal-Wallis test, logistic regression

**Table 2:** Summary of SSI classification results for basic lasso model (BLM), full lasso model (FLM), price penalized model (PPM), and XGBoost predictive model.

Model	CRP	BLM	BLM (*p*_max_=20)	FLM	FLM (*p*_max_=50)	PPM	PPM (*p*_max_=20)	XGBoost
Nr. features	4.4	35.6	12.3	32.6	**28.4**	25.2	12.1	
	[4.2, 4.6]	[33.1, 38.2]	[11.8, 12.9]	[30.2, 35.0]	[27.1, 29.6]	[22.9, 27.5]	[11.7, 12.6]	

AUC	0.797	0.947	0.943	0.954	**0.956**	0.952	0.951	0.954
	[0.788, 0.806]	[0.944, 0.951]	[0.940, 0.947]	[0.951, 0.958]	[0.953, 0.959]	[0.948, 0.955]	[0.948, 0.954]	[0.951, 0.957]

AUPRC	0.550	0.809	0.810	0.819	0.821	0.813	0.817	**0.829**
	[0.532, 0.568]	[0.798,0.821]	[0.799,0.822]	[0.807, 0.830]	[0.810, 0.832]	[0.801,0.824]	[0.805–0.828]	[0.818, 0.839]

Threshold	0.204	0.188	0.179	0.221	0.216	0.191	0.171	0.245
	[0.201–0.207]	[0.183–0.193]	[0.176–0.182]	[0.216–0.226]	[0.211–0.220]	[0.186–0.196]	[0.167–0.175]	[0.237–0.252]

Sensitivity	0.699	0.858	0.856	0.856	0.860	0.868	**0.874**	0.836
	[0.683, 0.715]	[0.846, 0.870]	[0.844, 0.868]	[0.845, 0.867]	[0.848, 0.871]	[0.857, 0.879]	[0.864, 0.885]	[0.824, 0.847]

Specificity	0.799	0.895	0.885	0.902	0.901	0.888	0.876	**0.919**
	[0.791, 0.808]	[0.891, 0.900]	[0.879, 0.891]	[0.896, 0.907]	[0.895, 0.906]	[0.882, 0.894]	[0.869, 0.882]	[0.915, 0.924]

PPV	0.469	0.673	0.654	0.688	0.687	0.663	0.641	**0.724**
	[0.454, 0.483]	[0.661, 0.684]	[0.640, 0.667]	[0.673, 0.703]	[0.673, 0.701]	[0.647, 0.678]	[0.628, 0.655]	[0.710, 0.739]

NPV	0.914	0.962	0.961	0.962	0.962	0.964	**0.966**	0.957
	[0.910, 0.919]	[0.958, 0.965]	[0.958, 0.964]	[0.958, 0.965]	[0.959, 0.966]	[0.961, 0.967]	[0.963, 0.969]	[0.954, 0.960]

**Table 3:** Evaluation of test data of SSI classification results using FLM for different *p*_max_ values.

Name	FLM, *p*_max_=10	FLM, *p*_max_=20	FLM, *p*_max_=30	FLM, *p*_max_=40	FLM, *p*_max_=50	FLM, *p*_max_=60	FLM, *p*_max_=70	FLM, *p*_max_=80	FLM, *p*_max_=90	FLM, *p*_max_=100	FLM, *p*_max_=No limit
Nr. features	4	10	21	29	29	40	37	34	33	44	34
AUC	0.966	**0.967**	0.961	0.961	0.961	0.961	0.961	0.962	0.961	0.962	0.962
AUPRC	**0.882**	0.872	0.880	0.874	0.874	0.875	0.873	0.873	0.871	0.877	0.874
Sensitivity	**0.920**	**0.920**	**0.920**	**0.920**	**0.920**	0.900	0.900	0.900	0.900	0.900	0.900
Specificity	0.871	0.865	0.893	0.893	0.893	**0.920**	0.910	0.900	0.900	**0.920**	0.900
PPV	0.667	0.657	0.701	0.701	0.701	0.780	0.763	0.734	0.726	0.763	0.726
NPV	**0.975**	**0.975**	**0.975**	**0.975**	**0.975**	0.971	0.970	0.970	0.970	0.970	0.970
Nr. predicted cases	69	70	65	65	65	59	61	62	62	59	62

**Table 4:** Upper half of the total number of features selected (above 50%) and their coefficient's signs in 100 repetitions of 10-fold cross-validation for FLM.

FLM variables	*N*	Sign
Leukocytes_nmbr_test_M	100	+
Sodium_nmbr_test_M	100	+
CRP_mean_M	99	+
Thrombocytes_prop_nmbr_test_M	99	+
Hemoglobin_prop_nmbr_test_S	97	−
Hemoglobin_prop_nmbr_test_M	97	+
Albumin_slope_L	96	−
Leukocytes_prop_nmbr_test_L	96	+
Albumin_mean_M	93	−
Amylase_prop_nmbr_test_L	93	+
Bilirubin.total_mean_M	91	+
CRP_prop_nmbr_test_S	88	−
Creatinine_slope_M	77	+
ALP_high_abn_prop_M	73	−
CRP_slope_L	73	+
Potassium_high_abn_prop_M	69	+
Creatinine_prop_nmbr_test_M	67	+
Hemoglobin_nmbr_test_M	56	+
Glucose_nmbr_test_M	55	+
ASAT_low_abn_prop_M	53	+
Albumin_slope_M	51	+

## Data Availability

The surgical site infection data recorded in the EHR at the Gastrointestinal Surgery Department in the University Hospital of North Norway used to support the findings of this study have not been made available because of the sensitive nature of data used in this study.

## Supplementary Materials

Supplementary Materials We provide a table with the blood test prices in Norwegian Kroner for the 14 most frequent tests over a 30-day period before surgery.
