# Supplementary material for: Maximizing Interpretability and Cost-Effectiveness of Surgical Site Infection (SSI) Predictive Models Using Feature-Specific Regularized Logistic Regression on Preoperative Temporal Data
Source: Comput Math Methods Med. 2019 Feb 19;2019:2059851. doi: 10.1155/2019/2059851 (PMC6399553; doi:10.1155/2019/2059851)
Supplement: Supplementary Materials — We provide a table with the blood test prices in Norwegian Kroner for the 14 most frequent tests over a 30-day period before surgery. [file 2059851.f1.pdf]

Kocbek et al. Maximizing interpretability and cost-effectiveness of Surgical Site Infection (SSI) predictive models using feature-specific regularized logistic regression on preoperative temporal data. *Computational and Mathematical Methods in Medicine*, 2019.

Supplementary Materials

Table S1: Blood test prices for 14 most frequent tests over a 30-day period before surgery

**Blood test**	**Price [Norwegian krone]**
Hemoglobin*	16.39
Leukocytes*	
Trombocytes*	
Amylase	13.72
CRP	10.94
Albumin	7.22
Kreatinin	7.16
Glukose	6.99
Bilirubin	6.63
ASAT	6.58
ALAT	6.55
ALP	6.38
Natrium	6.33
Kalium	6.22

*all 3 results obtained with Hematology Analyser
